# Updates in Stroke Treatment, Diagnostic Methods and Predictors of Outcome

**DOI:** 10.3390/jcm9092789

**Published:** 2020-08-29

**Authors:** Aristeidis H. Katsanos

**Affiliations:** Division of Neurology, McMaster University/Population Health Research Institute, 237 Barton St E, Hamilton, ON L8L 2X2, Canada; ar.katsanos@gmail.com; Tel.: +1-365-888-1441

## 1. Introduction

In recent years, there have been outstanding achievements in stroke diagnosis and care [1,2]. Our better understanding of the pathophysiological mechanisms and the advances in neuro-imaging have enabled us to diagnose stroke syndromes with remarkable precision and uncover underlying vessel pathologies that can be directly correlated with the stroke event [2]. Within a short period of time, endovascular thrombectomy (EVT) became the standard of care for patients with large vessel occlusions and symptom onset up to 24 h [3,4,5], while other recent trials introduced the use of perfusion imaging to guide intravenous thrombolysis in the extended time window [6].

This Special Issue of the Journal of Clinical Medicine features articles presenting considerations and improvements in acute stroke treatment, emerging neurosonology applications and novel predictors of stroke outcome.

### 1.1. Considerations and Improvements in Acute Stroke Reperfusion Therapies

Intravenous thrombolysis (IVT) in patients with a low National Institutes of Health Stroke Scale (NIHSS) score of 0–5 remains controversial. The Potential of rtPA for Ischemic Strokes with Mild Symptoms (PRISMS) trial was a phase 3, randomized, double-blind clinical trial that aimed to test the safety and efficacy of intravenous thrombolysis, with tissue plasminogen activator (tPA) administered within 3 h of symptom onset in acute ischemic stroke (AIS) patients with mild, non-disabling neurological deficits (baseline National Institutes of Health Stroke Scale (NIHSS) score equal to or less than 5) [7]. The study was prematurely terminated by the sponsor after the recruitment of 313 patients (one-third of the initially planned sample size) due to the low recruitment rates [7]. PRISMS investigators found comparable 3-month favorable functional outcomes between patients receiving IVT and aspirin, while highlighting an increased risk for symptomatic intracranial hemorrhage for patients randomized to intravenous tPA treatment [7].

Merlino et al. tested the hypothesis that the utility of intravenous tPA in patients with mild stroke symptoms may depend on their level of functional dependence at hospital admission [8]. Authors analyzed data form a prospectively collected database including 389 patients presenting with acute ischemic strokes and mild deficits [8]. Patients were stratified according to their baseline Barthel index (BI) score into those with functional dependence (BI score < 80) and those functionally independent (BI score ≥ 80) at baseline [8]. Merlino et al. found that intravenous thrombolysis with tPA was independently associated with more favorable functional outcomes at 3 months after stroke onset in patients that were judged to be dependent at hospital presentation [8]. The association between favorable 3-month outcomes and tPA administration was not evident for patients that were independent at their presentation, suggesting that the beneficial effect of tPA in patients with mild neurological syndromes on admission is influenced by their functional status at admission [8]. Despite the limitations of the present report, including the presence of unmeasured confounders due to the lack of randomization and bias by indication in the decision to deliver tPA, the authors address an important question that stroke physicians deal with in the everyday clinical practice. Until further studies provide compelling evidence for subgroups of patients with mild strokes for whom tPA treatment is futile or even harmful, eligible patients with disabling symptoms should not be excluded from prompt tPA administration.

Randomized controlled clinical trials have provided robust evidence on the benefit of both intravenous tissue tPA and EVT for improving the outcomes of acute ischemic stroke patients [9]. However, real world evidence is needed in order to unveil the real effectiveness of acute reperfusion stroke treatments in different countries and healthcare systems. Selection Criteria in Endovascular Thrombectomy and Thrombolytic Therapy (SECRET) is a prospective nationwide population multicenter registry that aims to explore the selection criteria and outcomes of patients who receive acute stroke reperfusion therapies in Korea [10]. SECRET investigators found significant increases in both the proportions of patients achieving successful recanalization (78.6% to 85.1%) and those discharged home (78.6% to 85.1%) from 2012 to 2017 [10]. A significant decrease in the time from hospital presentation to initiation of reperfusion therapy was also observed over the years 2012 to 2017 [10]. These data from Korea provide reassurance on the beneficial effect of reperfusion therapies in the real-world setting and the temporal improvements in treatment delivery. Population-based data are essential for the quality monitoring and optimal care delivery of acute reperfusion stroke therapies.

Rapid EVT delivery for acute ischemic stroke caused by large vessel occlusion leads to improved outcomes [11]. Optimizing intra-hospital management seems to be one of the most efficient ways to diminish treatment delays and decrease further the time from stroke onset to successful reperfusion. Direct transfer to the angiography suite, bypassing both the emergency department and the radiology room of the multidetector computed tomograph (CT), also known as one-stop management, has previously been suggested as an efficient method to reduce in-hospital delays [12]. In the angiography suite, patients with stroke symptoms receive a brain scan with the use of a flat-detector CT capable angiography-suite and, if eligible, they receive on the spot treatment with intravenous tPA and/or EVT [12]. Psychogios et al. examined if one-stop management can not only reduce intra-hospital treatment delays, but can also improve the functional outcomes of acute ischemic stroke patients with large vessel occlusion [13]. Large vessel occlusion was diagnosed in 72% and intravenous tPA was administered in 63% of the 230 total patients [13]. Compared to 43 case-matched patients triaged with multidetector CT, one-stop management was found to reduce the median door-to-reperfusion time by more than 40 min, and resulted in improved patient functional outcomes [13]. No safety concerns were noticed, as the incidence of intracranial bleeding and all-cause mortality was comparable between the two groups [13]. The authors correctly acknowledge in the limitations of their work that their findings might not be applicable to low volume stroke centers, as the number of patients with large vessel occlusions is expected to be much lower than the one described in the publication by Psychogios et al. [13]. The authors also state in their conclusion section that they have designed a prospective, randomized trial to evaluate the effectiveness and safety of their proposed one-stop protocol [13]. The results of this trial are expected to provide robust evidence on the optimal route selection for acute ischemic stroke patients with suspected large vessel occlusion and, if positive, change the flow of in-hospital stroke care delivery.

The underlying pathomechanism of a large vessel occlusion has been suggested to be associated with effectiveness of the EVT procedure and the outcomes of patients [14]. In a retrospective cohort study, Baek et al. investigated whether the status of leptomeningeal collaterals in the brain CT angiography can be a marker of the large vessel occlusion etiology [15]. Comparing leptomeningeal collateral patterns between patients with intracranial atherosclerotic disease and without, complete leptomeningeal collaterals were found to be more than two times more prevalent in patients with intracranial atherosclerotic disease [15]. Despite their fair negative predictive value, the presence of complete leptomeningeal collateral supply in baseline brain CT angiography was found to have an overall modest predictive value for the discrimination of the etiology of a large vessel occlusion [15]. The use of leptomeningeal collateral supply as a pre-procedural predictor for the stroke mechanism and outcomes of patients with large vessel occlusion treated with EVT deserves to be evaluated in multicenter prospective observational cohorts.

### 1.2. Emerging Applications of Neurosonology in Stroke

Although paroxysmal atrial fibrillation (AF) has been suggested to be present in at least a third of patients with cryptogenic stroke or transient ischemic attack (TIA) [16], current guidelines on secondary stroke prevention suggest that the clinical benefit of prolonged cardiac monitoring to uncover underlying paroxysmal AF after an acute ischemic stroke or TIA remains uncertain [17]. Identifying populations that might have a higher probability of underlying AF could lead to a more targeted use of prolonged cardiac monitoring for this patient population.

Liantinioti et al. sought to identify whether cardiac arrhythmia detection in spectral waveform analysis during neurosonology examinations with Carotid Duplex and Transcranial Doppler ultrasound may be associated with a higher likelihood of paroxysmal AF detection [18]. They evaluated 373 consecutive patients with recent cryptogenic strokes over a six-year period. The rate of AF detection on outpatient 24-h Holter ECG recording was 11% [18]. Arrhythmia detection during neurosonology evaluations was independently associated with a three-fold higher likelihood of PAF detection during follow-up [18]. The study by Liantinioti et al. highlights the importance of detecting and reporting cardiac arrhythmias during the neurosonology examination of patients with ischemic stroke, and further expands the utility of neurosonology in determining stroke etiology, by identifying those patients that have a higher probability of underlying paroxysmal AF and deserve more thorough cardiac rhythm monitoring. Given that antiplatelet treatment is known to confer inadequate protection, prompt anticoagulation in these patients can reduce the risk of future thromboembolic events [19].

Park et al. investigated the association between total carotid plaque number and long-term prognosis in ischemic stroke patients with AF [20]. Total plaque number was assessed with B-mode ultrasonography in 392 ischemic stroke patients with AF [20]. After a mean follow-up of 2.42 years, 28.8% of the patients suffered a major adverse cardiovascular event [20]. The presence of five plaques or more in the baseline carotid ultrasound was independently associated with an increased risk of both major adverse cardiovascular events and all-cause mortality [20]. Interestingly, the total plaque number along with the maximal plaque thickness and intima media thickness showed improved prognostic utility when added to the variables of the CHAD_2_DS_2_-VASc score [20]. Although Park et al. adjust their outcomes for baseline medications, it should be noted that there is a possibility for suboptimal medical management in their population, as 44% and 20% of patients were on antiplatelet and statin treatment, respectively, and despite the confirmed diagnosis of AF and history of stroke, only 22% patients were taking oral anticoagulant [20]. The findings by Park et al. suggest that patients with AF and high atherosclerotic burden are at increased risk for cardiovascular events. Carotid ultrasound could therefore serve as a valuable, non-invasive screening tool for identifying these high risk individuals.

CT perfusion imaging is used to guide systematic and endovascular stroke treatments for patients presenting in the extended time windows [4,5,6]. Ultrasound cerebral perfusion imaging techniques have been introduced and validated with different data acquisition and processing approaches [21]. In a very nice and comprehensive way, Eyding et al. summarize the evolution, different technical aspects and milestones in the use of cerebral ultrasound perfusion imaging over time [21]. The authors highlight future potential applications of cerebral ultrasound perfusion imaging as a bedside method of microcirculatory perfusion assessment [21]. Eyding et al. propose that in cases of the presence of sufficient temporal windows, a multi-modal approach for the detection of vessel occlusion, microvascular perfusion impairment or intracerebral hemorrhage is feasible with ultrasonographic techniques [21]. The evaluation of this method in the pre-hospital setting and the development of automated software algorithms are two directions that future research should focus on.

### 1.3. Novel Predictors of Stroke Outcome

Most physicians use their own clinical experience in predicting their patients’ outcomes after a stroke, as accurate prognostic models and tests of functional recovery in stroke patients are lacking to date [22].

Min et al. performed a prospective cohort study to evaluate the usefulness of interhemispheric functional connectivity as a predictor of motor recovery in stroke patients with unilateral severe upper-limb paresis [23]. Functional connectivity was measured in resting-state functional magnetic resonance imaging (MRI) scans at 1 month from stroke onset [23]. Good recovery, assessed with the Brunnstrom stage of upper-limb function at 6 months, was associated with higher functional connectivity values [23]. The authors conclude that interhemispheric functional connectivity measurement using resting-state functional MRI scans may provide useful clinical information for predicting hand motor recovery during stroke rehabilitation [23]. The predictive value of functional connectivity assessment for long-term motor outcomes in subacute stroke patients has to be confirmed by independent large-scale studies. Future studies need also to evaluate the applicability of resting-state functional MRI for the prediction of the recovery of stroke patients with non-motor deficits (e.g., visual defects) and strokes of afferent vascular distributions.

Ankle-brachial blood pressure index (ABI) is calculated by the ratio of the systolic blood pressure of an ipsilateral ankle divided by the higher systolic blood pressure of the two arms [24]. Low ABI has previously been reported to identify patients with symptomatic and asymptomatic peripheral arterial disease, and has been associated with a higher risk of early recurrent stroke in patients with an acute ischemic stroke and no history of symptomatic peripheral arterial disease [24]. Han et al. investigated the association of high ABI difference between the two arms and systolic blood pressure difference between the two ankles with short- and long-term outcomes in acute ischemic stroke patients without peripheral artery diseases [25]. After analyzing data from 2901 patients with acute ischemic stroke, followed over a median of 3.1 years, the authors found that a high ABI difference either between arms or ankles is associated with poor functional outcomes, long-term cardiovascular events and all-cause mortality [25].

The impact of hemoglobin status and red blood cell transfusions (RBC) on acute ischemic stroke outcomes is controversial. Kim et al. aimed to investigate whether RBC transfusions and hemoglobin variability affects the outcome of patients after an acute ischemic stroke [26]. The authors analyzed data from 2698 patients with acute ischemic stroke admitted in three tertiary hospitals. In total, 32 patients (4.9%) were transfused with packed RBCs during their admission [26]. Patients transfused more than 48 h after hospitalization were found to have a higher probability of poor outcomes at 3 months [26]. Hemoglobin variability during hospitalization, however, was not found to be associated with patient outcomes [26]. Despite the inherent limitations of the study design of Kim et al., it being a retrospective cohort study with limited power and high risk of bias due to the presence of unmeasured confounders, it raises the need for further research in order to elucidate the potential impact of blood transfusions timing on the outcomes of patients with recent acute ischemic stroke.

Although platelet activation and aggregation has been suggested to play an important role in the pathogenesis of ischemic stroke, the association between platelet reactivity and ischemic lesions is still debatable. Ischemic stroke patients with high on-treatment platelet reactivity have previously been reported to be at increased risk of recurrent cerebrovascular ischemic events [27]. Wisniewski et al. aimed to assess the relationship between platelet reactivity and the extent of ischemic cerebral lesions according to stroke etiology [28]. The evaluation of platelet reactivity was performed within 24 h after stroke onset in 69 patients [28]. An ischemic brain volume measurement was performed in MRI sequences at day 2–5 after stroke onset [28]. In the subgroup of patients with large-vessel disease, a correlation between platelet reactivity and acute ischemic core volume was evident in aspirin-resistant subjects [28]. Based on their findings, authors propose that in patients with ischemic stroke due to large-vessel disease, high on-treatment platelet reactivity affects the extent of ischemic lesions [28]. Apririn resistance among patients with stroke in the study by Wisniewski et al. was estimated at 31.8% and at 7%, with the methods of impedance and optical aggregometry [28].

In a subsequent publication in the same issue of the Journal of Clinical Medicine, Wisniewski et al. assessed the relationship of platelet reactivity with early and late prognosis after an acute ischemic stroke, according to the stroke etiology [29]. Performing platelet function testing with two aggregometric methods in 69 individuals, they found higher platelet reactivity in patients with severe neurological deficits on day 90 after stroke onset, when compared to the group of patients experiencing mild neurological deficits [29]. In patients with acute ischemic stroke attributed to large vessel disease, a significant correlation between the platelet reactivity and functional status on the first day was also uncovered, with patients resistant to aspirin having a significantly greater possibility of severe neurological deficits on the first day of stroke compared to their aspirin-sensitive counterparts [29].

Endothelial progenitor cells are considered to be a marker of both endothelial damage and endothelium regeneration ability [30]. In a third publication, Wisniewski et al. assessed the number of endothelial progenitor cells in patients with acute ischemic or hemorrhagic stroke, and evaluated whether there exist relationships with clinical status, radiological findings, risk factors, selected biochemical parameters and prognosis [30]. The number of endothelial progenitor cells was determined in serum on the first and eighth day after stroke onset using flow cytometry in 66 patients with lacunar ischemic stroke, 38 patients with hemorrhagic stroke, and 22 control subjects without acute cerebrovascular incidents [30]. Although a significantly higher number of endothelial progenitor cells on the first day of stroke compared to the control group was identified, no relationships between the number of endothelial progenitor cells in the acute phase of stroke and biochemical parameters, vascular risk factors or clinical condition were uncovered [30]. The authors concluded that endothelial progenitor cells are an early marker in acute stroke regardless of etiology, with no prognostic value being identified [30].

## 2. Conclusions

Although real-world evidence confirms the beneficial effect of reperfusion therapies in the real-world setting, as well as the temporal improvements in stroke treatment delivery over the last few years, several research areas are still pending further investigation. The utility of intravenous tPA in patients with non-disabling stroke syndromes, the use of ultrasound cerebral perfusion imaging in the pre-hospital setting and the optimal in-hospital route for acute ischemic stroke patients with suspected large vessel occlusion are topics in acute stroke care that need to be further investigated. Ongoing research also needs to focus on the development of reliable radiologic, clinical and biomarkers predictors of stroke outcome.

## References

[1] Baek J.-H., Kim B.M., Kim J.W., Kim D.J., Heo J.H., Nam H.S., Kim Y.D. (2020). Utility of Leptomeningeal Collaterals in Predicting Intracranial Atherosclerosis-Related Large Vessel Occlusion in Endovascular Treatment. J. Clin. Med..

[2] Katsanos A.H., Hart R.G. (2020). New horizons in pharmacologic therapy for secondary stroke prevention. JAMA Neurol..

[3] Rabinstein A.A. (2020). Update on treatment of acute ischemic stroke. Continuum Minneap Minn.

[4] Tsivgoulis G., Safouris A., Katsanos A.H., Arthur A.S., Alexandrov A.V. (2016). Mechanical thrombectomy for emergent large vessel occlusion: A critical appraisal of recent randomized controlled clinical trials. Brain Behav..

[5] Nogueira R.G., Jadhav A.P., Haussen D.C., Bonafe A., Budzik R.F., Bhuva P., Yavagal D.R., Ribo M., Cognard C., Hanel R.A. (2018). Thrombectomy 6 to 24 hours after stroke with a mismatch between deficit and infarct. N. Engl. J. Med..

[6] Albers G.W., Marks M.P., Kemp S., Christensen S., Tsai J.P., Ortega-Gutierrez S., McTaggart R.A., Torbey M.T., Kim-Tenser M., Leslie-Mazwi T. (2018). Thrombectomy for stroke at 6 to 16 hours with selection by perfusion imaging. N. Engl. J. Med..

[7] Tsivgoulis G., Katsanos A.H., Malhotra K., Sarraj A., Barreto A.D., Köhrmann M., Krogias C., Ahmed N., Caso V., Schellinger P.D. (2020). Thrombolysis for acute ischemic stroke in the unwitnessed or extended therapeutic time window. Neurology.

[8] Khatri P., Kleindorfer D.O., Devlin T., Sawyer R.N., Starr M., Mejilla J., Broderick J., Chatterjee A., Jauch E.C., Levine S.R. (2018). Effect of alteplase vs aspirin on functional outcome for patients with acute ischemic stroke and minor nondisabling neurologic deficits: The PRISMS randomized clinical trial. JAMA.

[9] Merlino G., Smeralda C., Lorenzut S., Gigli G.L., Surcinelli A., Valente M. (2020). To treat or not to treat: Importance of functional dependence in deciding intravenous thrombolysis of “mild stroke” patients. J. Clin. Med..

[10] Tsivgoulis G., Katsanos A.H., Alexandrov A.V. (2014). Reperfusion therapies of acute ischemic stroke: Potentials and failures. Front. Neurol..

[11] Kim Y.D., Heo J.H., Yoo J., Park H., Kim B.M., Bang O.Y., Kim H.C., Han E., Kim D.J., Heo J. (2020). Improving the clinical outcome in stroke patients receiving thrombolytic or endovascular treatment in korea: From the SECRET study. J. Clin. Med..

[12] Bourcier R., Goyal M., Liebeskind D.S., Muir K.W., Desal H., Siddiqui A.H., Dippel D.W.J., Majoie C.B., van Zwam W.H., Jovin T.G. (2019). Association of time from stroke onset to groin puncture with quality of reperfusion after mechanical thrombectomy: A meta-analysis of individual patient data from 7 randomized clinical trials. JAMA Neurol..

[13] Psychogios M.N., Behme D., Schregel K., Tsogkas I., Maier I.L., Leyhe J.R., Zapf A., Tran J., Bähr M., Liman J. (2017). One-stop management of acute stroke patients: Minimizing door-to-reperfusion times. Stroke.

[14] Wiśniewski A., Boinska J., Ziołkowska K., Lemanowicz A., Filipska K., Serafin Z., Ślusarz R., Rość D., Kozera G. (2020). Endothelial progenitor cells as a marker of vascular damage but not a predictor in acute microangiopathy-associated stroke. J. Clin. Med..

[15] Matias-Guiu J.A., Serna-Candel C., Matias-Guiu J. (2014). Stroke etiology determines effectiveness of retrievable stents. J. Neurointerv. Surg..

[16] Psychogios M.N., Maier I.L., Tsogkas I., Hesse A.C., Brehm A., Behme D., Schnieder M., Schregel K., Papageorgiou I., Liebeskind D.S. (2019). One-stop management of 230 consecutive acute stroke patients: report of procedural times and clinical outcome. J. Clin. Med..

[17] Sposato L.A., Cipriano L.E., Saposnik G., Ruνz Vargas E., Riccio P.M., Hachinski V. (2015). Diagnosis of atrial fibrillation after stroke and transient ischaemic attack: A systematic review and meta-analysis. Lancet Neurol..

[18] Kernan W.N., Ovbiagele B., Black H.R., Bravata D.M., Chimowitz M.I., Ezekowitz M.D., Fang M.C., Fisher M., Furie K.L., Heck D.V. (2014). Guidelines for the prevention of stroke in patients with stroke and transient ischemic attack: A guideline for healthcare professionals from the american heart association/american stroke association. Stroke.

[19] Liantinioti C., Palaiodimou L., Tympas K., Parissis J., Theodorou A., Ikonomidis I., Chondrogianni M., Zompola C., Triantafyllou S., Roussopoulou A. (2019). Potential utility of neurosonology in paroxysmal atrial fibrillation detection in patients with cryptogenic stroke. J. Clin. Med..

[20] Triantafyllou S., Katsanos A.H., Dilaveris P., Giannopoulos G., Kossyvakis C., Adreanides E., Liantinioti C., Tympas K., Zompola C., Theodorou A. (2020). Implantable cardiac monitoring in the secondary prevention of cryptogenic stroke. Ann. Neurol..

[21] Park H., Han M., Kim Y.D., Yoo J., Lee H.S., Choi J.K., Heo J.H., Nam H.S. (2019). Impact of the total number of carotid plaques on the outcome of ischemic stroke patients with atrial fibrillation. J. Clin. Med..

[22] Eyding J., Fung C., Niesen W.D., Krogias C. (2020). Twenty years of cerebral ultrasound perfusion imaging—Is the best yet to come?. J. Clin. Med..

[23] Jampathong N., Laopaiboon M., Rattanakanokchai S., Pattanittum P. (2018). Prognostic models for complete recovery in ischemic stroke: A systematic review and meta-analysis. BMC Neurol..

[24] Min Y.S., Park J.W., Park E., Kim A.-R., Cha H., Gwak D.-W., Jung S.-H., Chang Y., Jung T.-D. (2020). Interhemispheric functional connectivity in the primary motor cortex assessed by resting-state functional magnetic resonance imaging aids long-term recovery prediction among subacute stroke patients with severe hand weakness. J. Clin. Med..

[25] Tsivgoulis G., Bogiatzi C., Heliopoulos I., Vadikolias K., Boutati E., Tsakaldimi S., Al-Attas O.S., Charalampidis P., Piperidou C., Maltezos E. (2012). Low ankle-brachial index predicts early risk of recurrent stroke in patients with acute cerebral ischemia. Atherosclerosis.

[26] Han M., Kim Y.D., Choi J.K., Choi J., Ha J., Park E., Kim J., Song T.-J., Heo J.H., Nam H.S. (2020). Predicting stroke outcomes using ankle-brachial index and inter-ankle blood pressure difference. J. Clin. Med..

[27] Kim C., Lee S.H., Lim J.S., Oh M.S., Yu K.-H., Kim Y., Lee J.-H., Jang M.U., Jung S., Lee B.-C. (2020). Timing of transfusion, not hemoglobin variability, is associated with 3-month outcomes in acute ischemic stroke. J. Clin. Med..

[28] Fiolaki A., Katsanos A.H., Kyritsis A.P., Papadaki S., Kosmidou M., Moschonas I.C., Tselepis A.D., Giannopoulos S. (2017). High on treatment platelet reactivity to aspirin and clopidogrel in ischemic stroke: A systematic review and meta-analysis. J. Neurol. Sci..

[29] Wiśniewski A., Sikora J., Sławińska A., Filipska K., Karczmarska-Wódzka A., Serafin Z., Kozera G. (2020). High on-treatment platelet reactivity affects the extent of ischemic lesions in stroke patients due to large-vessel disease. J. Clin. Med..

[30] Wiśniewski A., Filipska K., Sikora J., Ślusarz R., Kozera G. (2020). The Prognostic Value of High Platelet Reactivity in Ischemic Stroke Depends on the Etiology: A Pilot Study. J. Clin. Med..

